# Evaluation of Renin–Angiotensin–Aldosterone System Components and Enzymes in Systemically Hypertensive Cats Receiving Amlodipine

**DOI:** 10.3390/ani13223479

**Published:** 2023-11-10

**Authors:** Darcy Adin, Clarke Atkins, Oliver Domenig, Catherine Glahn, Teresa DeFrancesco, Kathryn Meurs

**Affiliations:** 1 College of Veterinary Medicine, University of Florida, Gainesville, FL 32608, USA; 2 College of Veterinary Medicine, North Carolina State University, Raleigh, NC 27606, USAkate_meurs@ncsu.edu (K.M.); 3 Attoquant Diagnostics, 1110 Vienna, Austria; oliver.domenig@attoquant.com; 4 Coastal Cat Clinic, Pacifica, CA 94044, USA; cathy.glahn@thecoastalcatclinic.com

**Keywords:** feline, blood pressure, kidney

## Abstract

**Simple Summary:**

High blood pressure (hypertension) is a common medical issue in cats. Amlodipine is a medication used to treat hypertension, but it might activate hormone systems that can be harmful in the long term. This preliminary study evaluated a hormone system called the renin–angiotensin–aldosterone system (RAAS) in cats with high blood pressure being treated with amlodipine compared to healthy cats with normal blood pressure, and found that hypertensive cats treated with amlodipine were RAAS activated. Since RAAS activation can have harmful long-term effects, including cardiac fibrosis (scar formation in the heart) and salt and water retention, medications to suppress the RAAS might be helpful in cats with high blood pressure that are treated with amlodipine.

**Abstract:**

Background: Chronic renin–angiotensin–aldosterone system (RAAS) activation is harmful. Amlodipine activates RAAS in humans and dogs, but contradictory data exist for systemically hypertensive (SHT) cats. Hypothesis: Cats with SHT and chronic kidney disease treated with amlodipine (SHT/CKD-A) are RAAS activated. Animals: Client-owned cats: unmedicated normotensive (NT) cats (*n* = 9); SHT/CKD-A cats (*n* = 5) with median systolic blood pressure of 170 mmHg (vs. 195 mmHg, pre-treatment), chronic kidney disease, and receiving no RAAS-suppressive therapy. Methods: Serum was frozen (−80 °C) until RAAS analysis via equilibrium analysis. The RAAS variables (reported as median (minimum–maximum)) were compared between groups, using Mann–Whitney U test. Results: Angiotensin 1, angiotensin 1,7, angiotensin III, and angiotensin 1,5, and angiotensin-converting enzyme (ACE)-2 activity were higher in SHT/CKD-A cats compared to NT cats, while ACE activity was lower in SHT/CKD-A cats compared to NT cats (*p* < 0.05 all). A marker for alternative RAAS influence (ALT-S) was significantly higher (69; 58–73 pmol/pmol) in SHT/CKD-A cats compared to NT cats (35; 14–63 pmol/pmol; *p* = 0.001). Aldosterone concentrations were significantly higher (393; 137–564 pmol/L) in SHT/CKD-A cats compared to NT cats (129; 28–206 pmol/L; *p* = 0.007). Conclusion and Clinical Importance: Circulating RAAS is activated in systemically hypertensive cats receiving amlodipine. Although this study did not parse out the individual contributions of SHT, chronic kidney disease, and amlodipine, the findings suggest that the use of concurrent RAAS-suppressant therapy, specifically aldosterone antagonism, in amlodipine-treated SHT cats with chronic kidney disease might be indicated.

## 1. Introduction

Systemic hypertension (SHT) is important because of associated target organ damage that affects the arterioles, kidneys, eyes, heart, and central nervous system [1,2]. An incompletely understood combination of barotrauma and the neurohormonal response to hypertension, related kidney disease, and even therapeutic efforts results in target organ damage [3,4]. Blood pressure control with beta-blockers, diuretics and angiotensin-converting enzyme (ACE) inhibitors alone, or in combination, is only modestly effective [5]. The introduction of amlodipine, a direct arteriolar vasodilator, profoundly improved the management of SHT in cats [6,7]. Recently, telmisartan, an angiotensin receptor blocker, has also proven to be efficacious in SHT cats [8,9].

Potentially harmful effects of amlodipine have been demonstrated in human SHT [3,10] and in normal dogs [4]. This dihydropyridine calcium channel-blocking vasodilator not only lowers blood pressure, but also activates the renin–angiotensin–aldosterone system (RAAS) and the sympathetic nervous system [3,4]. Therefore, while lowering BP, amlodipine might promote target organ damage due to neurohormonal discharge. Supportive of this possibility, one study found that an equal reduction in blood pressure in people with SHT using beta-blockade but without RAAS suppression did not produce a regression of SHT-associated anatomical and physiological arteriolar dysfunction, while beta-blockade with concurrent RAAS suppression did [11]. Published findings regarding the status of the RAAS for cats with SHT treated with amlodipine are conflicting, with one study showing no increase in aldosterone despite renin elevation [12] and another study showing global RAAS activation in SHT treated with amlodipine but not in untreated SHT cats [13].

The objective of this preliminary study was to comprehensively evaluate RAAS metabolites and enzymes in amlodipine-treated systemically hypertensive cats with chronic kidney disease (SHT/CKD-A) using equilibrium analysis. Our study was initiated before the recent publication evaluating the RAAS in cats with SHT, which utilized the same approach to RAAS analysis [13]. We hypothesized that naturally SHT cats with chronic kidney disease receiving chronic amlodipine therapy would show RAAS activation compared to NT cats.

## 2. Methods

The procedures for this preliminary study were approved by the Institutional Animal Care and Use Committee of North Carolina State University # 17-063-O, and informed client consent was obtained. Included NT cats were from North Carolina State University and were determined to be healthy through patient history, physical examination, laboratory testing (complete blood count, biochemistry profile, thyroid testing if indicated), blood pressure measurement (<160 mmHg), and echocardiography. Cats with SHT/CKD-A cats were from a feline veterinary hospital (Coastal Cat Clinic, Pacifica, CA, USA) and had a baseline blood pressure >170 mmHg that was treated with amlodipine but not RAAS-suppressive medications (i.e., ACE-inhibitors, angiotensin-receptor blockers, spironolactone were not allowed). The diagnosis of SHT was based on the repeated finding of blood pressure >170 mmHg on more than one visit and supported by fundic examination when possible. Systolic blood pressure was measured using Doppler technology with a cuff that was 40% of the tail or forelimb used, after a 5–10 min period of acclimatization. At least 5 blood pressure readings were obtained and averaged, discarding the highest and lowest values. Cats were assessed for systemic diseases using complete blood count, serum biochemistry panel, and thyroid testing. Renal disease was staged using the International Renal Interest Society recommendations (http://www.iris-kidney.com/pdf/IRIS_Staging_of_CKD_modified_2019.pdf (accessed 16 March 2020)) using bloodwork (blood urea nitrogen, serum creatinine, symmetric dimethylarginine, and urine-specific gravity). Three milliliters of blood were collected from a peripheral vein and centrifuged to a separate serum, which was frozen at −80 °C until batched quantification of RAAS components.

### 2.1. Equilibrium Analysis of RAAS Components

The equilibrium concentrations of 6 different angiotensin peptide metabolites and aldosterone in serum samples were quantified, as described previously, using LC-MS/MS at a service-providing laboratory (Attoquant Diagnostics, Vienna, Austria) [14,15]. The lower limit of quantification was 3.0 pmol/L for angiotensin I, 2.0 pmol/L for angiotensin II, 3.0 pmol/L for angiotensin 1,7, 2.0 pmol/L for angiotensin 1,5, 2.5 pmol/L for angiotensin III, 2.0 pmol/L for angiotensin IV, and 15.0 pmol/L for aldosterone. The ratio of angiotensin II to angiotensin I was calculated as a marker for ACE activity (ACE-S) [16]. Angiotensin I and angiotensin II were summed as a marker for plasma renin activity (PRA-S) [17]. The ratio of aldosterone to angiotensin II (AA2) was calculated as an indicator of adrenal responsiveness to angiotensin II-induced stimulation of aldosterone release [18]. Renin-independent alternative RAAS activation (ALT-S) was calculated, using the formula ([angiotensin 1,7 + angiotensin 1,5]/[angiotensin I + angiotensin II + angiotensin 1,7 + angiotensin 1,5]) [19].

### 2.2. Enzyme Activity

The activities of ACE, ACE2 and chymase were measured with a classical kinetics approach using spiked substrate, applying the natural substrate (e.g., ex vivo spiked angiotensin I for ACE activity and chymase activity, and angiotensin II for ACE2 activity) [1,15]. Activities of ACE and chymase were determined by measuring the formation of angiotensin II after addition of angiotensin I in the presence and absence of an ACE inhibitor and a chymase inhibitor to determine the ACE-inhibitor-sensitive and chymase-inhibitor-sensitive fractions of angiotensin II generation. The inhibitor-sensitive ACE and chymase-specific turnover was converted to an ACE and chymase activity using a calibration curve of recombinant human ACE or chymase (Bio-Techne, Wiesbadan-Nordentadt, Germany), respectively.

ACE2 activity was assayed by measuring the turnover of angiotensin II to angiotensin 1,7 with and without an ACE2 inhibitor (MLN-4760). The inhibitor-sensitive ACE2-specific turnover was converted to an ACE2 activity using a calibration curve of recombinant human ACE2 (Bio-Techne, Wiesbadan-Nordentadt, Germany).

### 2.3. Statistical Analysis

Sample size calculations were not performed a priori because of the exploratory nature of the study. Statistical analysis was performed using commercially available software (GraphPad Prism 8, San Diego CA, USA). Data were tested for normality using the Kolmogorov–Smirnov test and are reported as median and range (minimum–maximum) because of small sample size and non-normal distribution for some variables. Graphical representation of median values of RAAS metabolites and their relationship to each other through enzymes was created with Microsoft Office. Demographics and RAAS results from SHT/CKD-A cats and NT cats were compared using the Mann–Whitney U test. Sex distribution was compared between groups using Fisher’s exact test. Values below the lower limit of assay quantification were calculated as ½ the lower limit of detection for statistical analysis only. The relationship of ACE2 to ALT-S and to ACE was explored with Spearman’s correlation coefficient in all cats. Significance was set at *p* < 0.05.

## 3. Results

Nine NT cats (four male—castrated, five female—spayed) and 5 SHT/CKD-A cats (three male—castrated, two female—spayed) were included in this study. The NT cats were 7.2 (4–9) years and weighed 5.6 (4.4–8.2) kg. All cats were healthy, without evidence of anemia, infection, azotemia, cardiac disease, diabetes, hypertension, hyperthyroidism, or other medical diseases. The blood pressure for NT cats was 142 (100–160) mmHg.

The SHT/CKD-A cats were 17 (15–19) years and weighed 3.6 (3.2–4.3) kg and were older and weighed less than the NT cats (*p* = 0.001 both). No sex differences were present between groups (*p* = 1.0). The SHT/CKD-A cats had chronic kidney disease, IRIS stage 2 or 3 (blood urea nitrogen median 44, range 29–71 mg/dL, serum creatinine median 3.0, range 1.8–3.3 mg/dL, symmetric dimethylarginine median 14, range 12–16 µg/dL, and urine-specific gravity median 1.016, range 1.005–1.022). Pre-treatment blood pressure for SHT/CKD-A cats was 195 (170–200) mmHg. All SHT/CKD-A cats received amlodipine at a total daily dosage of 0.29 (0.17–0.70) mg/kg for a duration of 1.5 (0.5–3) years. Blood pressure was considered to be well-controlled for three cats (130, 142, and 148 mmHg) and poorly controlled for two cats (170 and 180 mmHg). Two cats also received methimazole for hyperthyroidism (one controlled with a total T4 of 1.3 µg/dL and one uncontrolled with an average T4 close to the time of sampling of 7.2 µg/dL) and one received insulin for diabetes mellitus. Only one SHT/CKD-A cat was fed a renal diet, which was only a part of its food intake.

**Figure 1:**
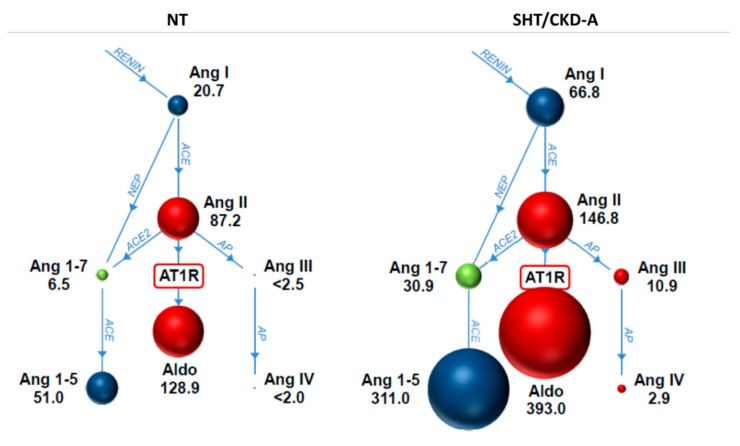
Serum renin–angiotensin–aldosterone system metabolites are shown for normotensive (NT) cats (*n* = 9) and systemically hypertensive cats with chronic kidney disease treated with amlodipine (SHT/CKD-A) (*n* = 5). The size of the spheres is proportional to the median concentration of the peptides shown beside the sphere. Enzymes catalyzing metabolite conversions are shown as arrows in light blue. Ang, Angiotensin; ACE, Angiotensin-converting enzyme; NEP, neutral endopeptidase; AP, aminopeptidase; AT1R, angiotensin receptor Type 1; Aldo, Aldosterone.

Except for ACE activity and ACE-S, which were significantly lower in SHT/CKD-A compared to NT cats, all other RAAS variables were higher in SHT/CKD-A cats compared to NT cats, with angiotensin I, angiotensin 1,7, angiotensin 1,5, angiotensin III, aldosterone, ACE2, and ALT-S showing statistical significance (Table 1). Figure 1 graphically depicts the global RAAS activation in SHT/CKD-A cats compared to NT cats.

**Figure 2:**
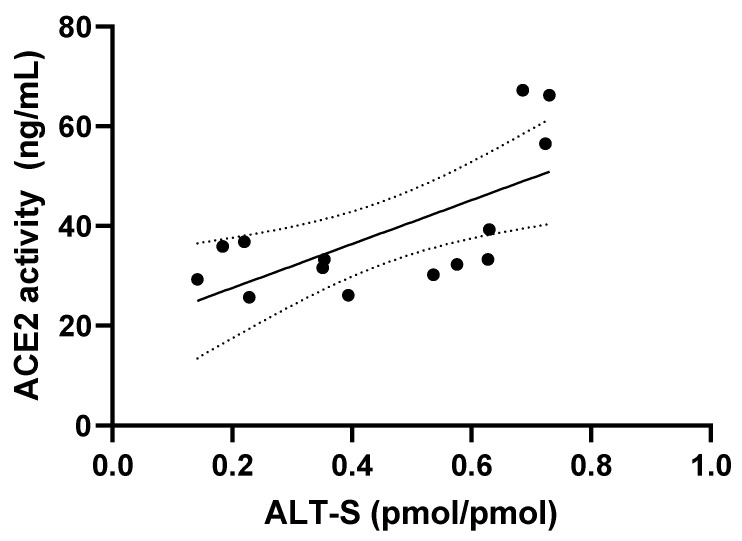
The activities of ACE2 and the ALT-S, an alternative RAAS activation marker calculated as (angiotensin 1,7 + angiotensin 1,5)/(angiotensin I + angiotensin II + angiotensin 1,7 + angiotensin 1,5) for all cats in the study are shown, depicting a moderately positive correlation (r_s_ = 0.62, *p* = 0.02); 95% confidence intervals (dotted line) and the line of best fit (solid line) are shown.

**Figure 3:**
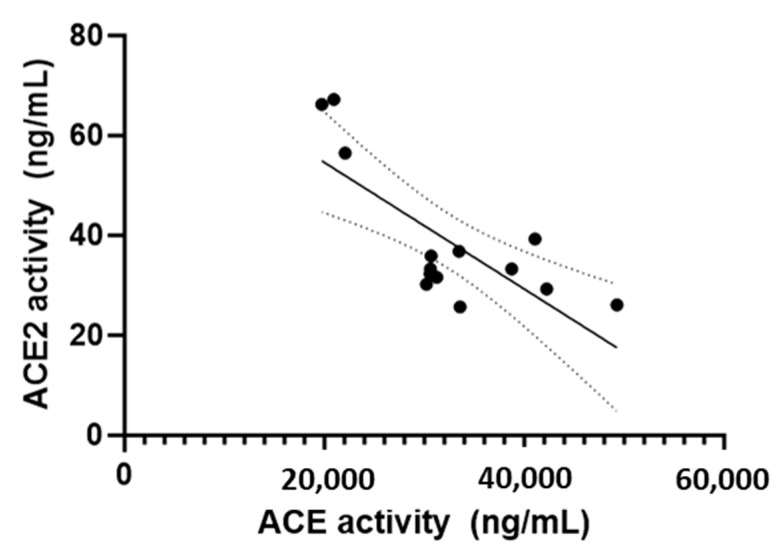
The activities of ACE and ACE2 for all cats in the study are shown, depicting a moderately negative correlation (r_s_ = −0.59, *p* = 0.03); 95% confidence intervals (dotted line) and the line of best fit (solid line) are shown.

The activity of ACE2 and ALT-S were significantly and moderately positively correlated (r_s_ = 0.62, *p* = 0.02) (Figure 2). The activities of ACE2 and ACE were significantly and moderately negatively correlated (r_s_ = −0.59, *p* = 0.03) (Figure 3).

## 4. Discussion

We found global RAAS activation in SHT/CKD-A cats in this preliminary study, with both classical RAAS components (e.g., angiotensin I, angiotensin III, and aldosterone) and alternative RAAS components (e.g., angiotensin 1,7, angiotensin 1,5, ALT-S) being significantly higher in SHT/CKD-A cats compared to NT cats. Classical RAAS metabolites mediate vasoconstriction, sodium retention, and inflammation, while alternative RAAS metabolites mediate vasodilation, natriuresis, and anti-inflammation [20]; the overall balance and importance of classical and alternative RAAS influences in SHT/CKD-A cats is uncertain in light of both arms of the RAAS cascade being enhanced. Our results are similar to those of Ward et al., which showed global RAAS activation in cats with systemic hypertension being treated with amlodipine compared to untreated cats with systemic hypertension as well as healthy cats [13]. Although RAAS activation in SHT/CKD-A cats in our study was global, a calculated measure of alternative RAAS (ALT-S) was higher in SHT/CKD-A cats compared to NT cats, which suggests that the alternative, beneficial metabolite influence might be greater than the classical, maladaptive metabolite influence in SHT/CKD-A cats. The significant correlation between ALT-S and ACE2 activity is expected because ACE2 is the major enzyme facilitating the production of alternative metabolites [20,21]. The contribution of other enzymes to angiotensin 1,5 and angiotensin 1,7 production (such as neutral endopeptidase) was not evaluated in this study.

Although ALT-S provides an estimate of the balance between alternative angiotensin metabolites compared to classical angiotensin metabolites, this calculation does not account for aldosterone, which is maladaptive through the promotion of sodium and water retention and myocardial fibrosis [20]. Despite the predominance of alternative (beneficial) angiotensin metabolite production in SHT/CKD-A cats, aldosterone was higher in SHT/CKD-A cats compared to NT cats, which indicates that undesirable effects mediated by aldosterone, including fibrosis, sodium retention, and potassium excretion, could occur in these cats. These findings are also in line with that of a previous study [13], which showed that amlodipine treatment was a predictor of increased RAAS biomarker concentrations, but contradictory to another study that failed to demonstrate aldosterone elevations in cats with hypertension [12]. Differences between studies might be due to different methods for assessing aldosterone concentrations or to different study populations. The results of our study suggest that aldosterone antagonism with medications such as spironolactone have a basis for potential clinical benefits in the treatment of cats with systemic hypertension using amlodipine.

Higher angiotensin II in SHT/CKD-A cats did not reach statistical significance when compared to NT cats, but this might be due to the greater ACE2 activity in SHT/CKD-A cats, which would reduce the concentration of this major ACE2 substrate (angiotensin II). Less-than-expected increases in angiotensin II might also have been due to a reduced ACE activity in SHT/CKD-A cats, which has also been previously shown in renal tissues [22]. Study power might have also been too low to detect group differences in angiotensin II, which were numerically but not statistically significantly higher in SHT/CKD-A cats compared to NT cats, especially in light of the high measurement variability. Even though there were no significant angiotensin II group differences, aldosterone concentrations were significantly higher in SHT/CKD-A cats compared to NT cats, similar to a previous study [13]. The major source of aldosterone production is from aldosterone synthase, which can be stimulated by angiotensin II, potassium concentrations, and adrenocortical-stimulating hormone; however, other sources, such as sympathetic nervous system stimulation, are also possible and could have impacted the aldosterone concentrations in this study [23,24]. These other potential sources were not evaluated in this study, but the lack of statistically significant group differences for AA2 (an estimate of adrenal responsiveness to angiotensin II) suggests other contributing sources of aldosterone stimulation. Circulating chymase was not different between groups, so this enzyme likely did not impact angiotensin II formation differently for NT and SHT/CKD-A cats.

We found a reciprocal relationship between ACE and ACE2 for all cats in this study. Similarly, an inverse relationship between these ACE homologs has been reported in a model of RAAS activation in rats [25], raising the possibility that their relationship might serve to regulate the production of metabolites produced by the cascade when global RAAS activation is present. Angiotensin II has been shown to differentially regulate the expression of ACE, as well as ACE2 expression and activity in spontaneously hypertensive mice [26], emphasizing the complexity of the system and the feedback mechanisms that could impact the metabolite concentrations and enzyme activities. Hypertension was variably controlled for the cats of this study; therefore, suboptimal control or the underlying stimulus for hypertension (e.g., chronic kidney disease for all of the SHT/CKD-A cats) could still regulate the expression of these enzymes [22,27]. Alternatively, there might be other regulators of these enzymes such as gene polymorphisms, other diseases, breed, sex, etc. [28,29,30]. The lack of chymase differences between groups suggests that the lower ACE activity in SHT/CKD-A cats did not stimulate circulating chymase up-regulation; however, tissue chymase activity was not evaluated. Lourenco et al. reported a similar finding (reduced renal tissue ACE mRNA) in cats with chronic kidney disease, which suggested ACE downregulation in this disease [22]. The ratio of angiotensin II to angiotensin I (ACE-S) in SHT/CKD-A cats was low, similar to the direct measurement of ACE activity in our study, further supporting ACE as the major creator of angiotensin II, with minimal chymase contribution [16].

This study has strengths and weaknesses that should be considered during interpretation. A strength of this study is the inclusion of circulating RAAS enzyme activities (ACE, ACE2, and chymase), which have not previously been reported in this feline disease. Knowledge of enzyme activities is useful for the interpretation of metabolite concentrations and relationships, but further studies are needed to understand the regulators of these enzymes. A significant limitation of this study is that the groups were not matched for age and weight, which could have impacted the results. The small sample size and lack of a priori sample size calculation are other important limitations of this study, which might have affected our ability to find group differences for some metabolites. Even with the small sample size, however, group differences were found for many RAAS metabolites, calculations, and enzymes. It is possible that concurrent chronic metabolic or hormonal diseases for some of the SHT/CKD-A cats (especially the one cat with sub-optimally controlled hyperthyroidism) might have impacted our results, because one study showed reductions in renin and aldosterone in hyperthyroid cats after treatment [31]. All of the SHT/CKD-A cats in this study had an underlying kidney disease, which might be associated with RAAS alterations. However, the study by Ward et al. [13] did not find a difference in RAAS variables between untreated systemically hypertensive cats (the majority of which had chronic kidney disease and two of which had hyperthyroidism) and healthy cats, lending support to the changes in treated systemically hypertensive cats being related to amlodipine. Another limitation of this study was the lack of an untreated SHT group of cats with chronic kidney disease, as well as the lack of pre-treatment sampling. The similar study by Ward et al., however, showed that the RAAS profile of untreated SHT cats, most of whom had chronic kidney disease, was not different from that of healthy cats [13]. This study [13] suggested that amlodipine treatment was the major stimulus for RAAS activation in that population of hypertensive cats, and our study results support this finding. Although amlodipine is considered an effective treatment for systemic hypertension in cats [2,6,7,32], RAAS activation, and specifically aldosterone production, associated with the medication or resultant vasodilation could promote target organ damage, warranting concurrent RAAS suppressive therapy. Further study is needed to investigate the role of aldosterone antagonism in this population.

## 5. Conclusions

The results of this preliminary study support the findings of, and provide additive information to other studies [13,22] in demonstrating global RAAS activation in cats with systemic hypertension and chronic kidney disease that are treated with amlodipine. The importance of the apparently greater alternative angiotensin metabolite presence, likely mediated by a higher ACE2 activity in SHT/CKD-A cats compared to NT cats, is uncertain in light of higher aldosterone concentrations in SHT/CKD-A cats. Although amlodipine is an effective anti-hypertensive medication, the results from our study and others suggest that concurrent RAAS suppressive therapy might be indicated (especially aldosterone antagonism) to mitigate the potentially maladaptive effects of hyperaldosteronism.

## Figures and Tables

**Table 1:** Median (minimum–maximum) measured and calculated variables for the renin–angiotensin–aldosterone system are shown for normotensive (NT) cats and cats with systemic hypertension treated with amlodipine (SHT/CKD-A).

Variable	NT Cats (*n* = 9)	SHT/CKD-A Cats (*n* = 5)	*p* Value
PRA-S (pmol/L)	108 (34–206)	199 (101–335)	0.06
angiotensin I (pmol/L)	21 (2–36)	67 (34–151)	**0.002**
angiotensin II (pmol/L)	87 (30–177)	147 (67–184)	0.11
angiotensin 1,7 (pmol/L)	6.5 (1.3–11.7)	30.9 (17.8–70.5)	**0.001**
angiotensin 1,5 (pmol/L)	51 (16–135)	311 (243–805)	**0.001**
angiotensin III (pmol/L)	1.3 (1.3–10.0)	10.9 (5.4–14.7)	**0.003**
angiotensin IV (pmol/L)	1.3 (1.3–4.4)	2.9 (2.2–3.1)	0.21
aldosterone (pmol/L)	129 (28–206)	393 (137–564)	**0.007**
AA2 (pmol/pmol)	1.2 (0.2–3.3)	2.7 (0.8–7.6)	0.24
ACE-S (pmol/pmol)	5.4 (1.9–9.8)	2.0 (1.1–3.0)	**0.007**
ACE activity (ng/mL)	33.6 (30.2–49.3)	22.1 (19.8–30.6)	**0.004**
ACE2 activity (ng/mL)	32 (26–39)	57 (32–67)	**0.045**
chymase activity (ng/mL)	0.3 (0.1–2.0)	0.8 (0.2–1.8)	0.44
ALT-S (pmol/pmol)	35 (14–63)	69 (58–73)	**0.001**

PRA-S; plasma renin activity marker (angiotensin I + angiotensin II), AA2; ratio of aldosterone to angiotensin II as a marker of adrenal responsiveness, ACE-S; angiotensin-converting enzyme marker (angiotensin II/angiotensin I), ACE2; angiotensin-converting enzyme 2 activity, ALT-S; alternative renin–angiotensin system activation marker (angiotensin 1,7 + angiotensin 1,5)/(angiotensin I + angiotensin II + angiotensin 1,7 + angiotensin 1,5). *p* < 0.05 is emboldened.

## Data Availability

The raw data presented in this study are available in Supplementary Table S1.

## Supplementary Materials

The following supporting information can be downloaded at: https://www.mdpi.com/article/10.3390/ani13223479/s1, Table S1: Raw data.

## Abbreviations

AA2	adrenal responsiveness marker (aldosterone/angiotensin II)
ACE	angiotensin-converting enzyme
ACE-S	ACE activity marker (angiotensin II/angiotensin I)
ALT-S	alternative RAAS activation marker ([angiotensin 1,7 + angiotensin 1,5]/[angiotensin I + angiotensin II + angiotensin 1,7 + angiotensin 1,5])
BP	blood pressure
LC-MS/MS	liquid chromatography–tandem mass spectrometry
NT	normotensive
PRA-S	plasma renin activity marker (angiotensin I + angiotensin II)
RAAS	renin–angiotensin–aldosterone system
SHT	systemic hypertension
SHT/CKD-A	systemic hypertension with chronic kidney disease treated with amlodipine
